# Presentation of Smoking-Associated Cues Does Not Elicit Dopamine Release after One-Hour Smoking Abstinence: A [^11^C]-(+)-PHNO PET Study

**DOI:** 10.1371/journal.pone.0060382

**Published:** 2013-03-29

**Authors:** Lina Chiuccariello, Isabelle Boileau, Mihail Guranda, Pablo M. Rusjan, Alan A. Wilson, Laurie Zawertailo, Sylvain Houle, Usoa Busto, Bernard Le Foll

**Affiliations:** 1 Translational Addiction Research Laboratory, Centre for Addiction and Mental Health (CAMH), Toronto, Canada; 2 Campbell Family Mental Health Research Institute, Centre for Addiction and Mental Health (CAMH), Toronto, Canada; 3 Addiction Imaging Research Group, Centre for Addiction and Mental Health (CAMH), Toronto, Canada; 4 Research Imaging Center, Centre for Addiction and Mental Health (CAMH), Toronto, Canada; 5 Clinical Neuroscience Program, Centre for Addiction and Mental Health (CAMH), Toronto, Canada; 6 Alcohol Research and Treatment Clinic, Centre for Addiction and Mental Health (CAMH), Toronto, Canada; 7 Department of Psychiatry, University of Toronto, Toronto, Canada; 8 Department of Pharmacology and Toxicology, University of Toronto, Toronto, Canada; 9 Department of Family and Community Medicine, University of Toronto, Toronto, Canada; 10 Faculty of Pharmacy, University of Toronto, Toronto, Canada; 11 Institute of Medical Sciences, University of Toronto, Toronto, Canada; Goethe University Frankfurt, Germany

## Abstract

The presentation of drug-associated cues has been shown to elicit craving and dopamine release in the striatum of drug-dependent individuals. Similarly, exposure to tobacco-associated cues induces craving and increases the propensity to relapse in tobacco- dependent smokers. However, whether exposure to tobacco-associated cues elicits dopamine release in the striatum of smokers remains to be investigated. We hypothesized that presentation of smoking-related cues compared to neutral cues would induce craving and elevation of intrasynaptic dopamine levels in subregions of the striatum and that the magnitude of dopamine release would be correlated with subjective levels of craving in briefly abstinent tobacco smokers. Eighteen participants underwent two [^11^C]-(+)-PHNO positron emission tomography (PET) scans after one-hour abstinence period: one during presentation of smoking-associated images and one during presentation of neutral images. Smoking cues significantly increased craving compared to neutral cues on one, but not all, craving measures; however, this increase in craving was not associated with overall significant differences in [^11^C]-(+)-PHNO binding potential (BP_ND_) (an indirect measure of dopamine release) between the two experimental conditions in any of the brain regions of interest sampled. Our findings suggest that presentation of smoking cues does not elicit detectable (by PET) overall increases in dopamine in humans after one-hour nicotine abstinence. Future research should consider studying smoking cue-induced dopamine release at a longer abstinence period, since recent findings suggest the ability of smoking-related cues to induce craving is associated with a longer duration of smoking abstinence.

## Introduction

Smoking is the leading preventable cause of morbidity and mortality in Western Society. Although a majority of smokers express the desire to quit smoking, most are unsuccessful [1], [2]. Exposure to contextual-cues associated with drug-use induces craving [3], [4], [5], [6] and increases the propensity to relapse in drug users [7], [8], [9], [10]. Understanding the mechanism of cue-induced relapse is essential in developing evidence-based treatment strategies that could reduce the impact of cue-induced cravings in smokers.

Dopamine (DA) neurons, shown to respond to reward (drug or other) and to “conditioned” predictors of reward, are believed to be involved in response to drug-cues [11]. Specifically, DA neuron firing and DA release measured with microdialysis or voltametry have been associated with response to drug associated cues in most [12], [13], [14], [15], [16] though not all [17] animal studies. Positron Emission Tomography (PET) has allowed the investigation of the role of DA in response to conditioned cues in humans *in vivo* [6], [12], [18], [19], [20], [21], [22], [23].

PET is versatile and a minimally invasive technique that can be used to assess dopaminergic response to a pharmacological or non-pharmacological challenge (such as conditioned stimuli presentation) in humans [24]. In this regard, PET studies have shown DA release in sub-compartments of the striatum of both cocaine [6], [22] and opiate [21] addicted individuals in response to drug-related imagery, which was related to addiction severity and drug craving [6], [22]. fMRI as well as FDG-PET studies have echoed some of these findings by showing activation of reward associated regions during presentation of drug-related cues to drug addicted individuals [4], [10], [25], [26], [27], [28], [29], [30], [31], [32]. Despite the evidence from animal models of addiction, and neuroimaging studies suggesting the importance of DA in cue-induced drug-seeking and craving, no research to date has investigated the dopaminergic response to presentation of smoking-related cues compared to neutral cues in tobacco smokers.

In this study we used PET and the DA D_2/3_ agonist radioligand [^11^C]-(+)-PHNO [33], in combination with a validated smoking cue-paradigm [34], [35] to test the hypothesis that presentation of smoking-related images would elicit cravings and would increase DA release in the striatum and that the two phenomena would be related. Our choice of using [^11^C]-(+)-PHNO (vs. [^11^C]raclopride) was motivated by the finding that [^11^C]-(+)-PHNO has a higher displacement potential relative to [^11^C]raclopride and therefore may be more sensitive to detect acute fluctuations in DA release induced by smoking-related conditioned cues [36], [37], [38].

## Materials and Methods

### Ethics Statement

This study was approved by the Centre for Addiction and Mental Health (CAMH) Research Ethics Board in accordance with the Declaration of Helsinki. Prior to participation in this study and following a detailed explanation of the study protocol, all participants provided written informed consent.

### Study Participants

Participants between the ages of 18–45 were recruited by advertisement in local newspapers and on-line postings. They were non treatment-seeking regular smokers of ≥10 cigarettes per day, for at least the past two years and scored greater ≥4 on the Fagerstrom Test for Nicotine Dependence (FTND) [39]. Participants did not meet criteria for abuse or dependence on any other drug of abuse and tested negative at screening on a broad-spectrum gas chromatography and mass spectroscopy urine drug toxicology screen for drugs of abuse, including cannabis. Specific exclusion criteria included pregnancy, current medication use, claustrophobia, cardiovascular or cerebrovascular disease, any Axis I psychiatric disorder (other than nicotine dependence) as determined by the Mini International Neuropsychiatric Interview (MINI) [40], a history of neurological illness/head trauma, learning disabilities, and the presence of metal objects in the body. Participants were not told to abstain from smoking but were asked not to consume caffeine on the day of the PET scan.

### Experimental procedure

Subjects were invited to take part in a PET study, which took place on two separate days, at least one week apart. On each scan day, upon arrival at the CAMH lab, subjects were instructed to smoke one cigarette of their preferred brand one hour before the scan. Following this, expired carbon monoxide (CO) (Micro III Smokerlyzer, Bedfont Instruments, Kent, England) was measured. This interval was chosen to minimize withdrawal symptoms that may lead to a ceiling effect on self-reported craving and mask cue-induced subjective changes [41]. Subjects were also told that they should expect to smoke one hour after the scan to control for expectancy, which could affect DA release [30], [42], [43].

One hour after smoking, subjects were placed in a supine position inside the PET camera and fitted with the thermoplastic head fixation system to minimize movement (Orfit Industries, USA). Scans were performed using a PET/CT camera system (Siemens Medical Imaging, Knoxville TN), which measures radioactivity in 81 trans axial slices with a reconstructed pixel size of 1.07×1.07×2.0 mm each with an in-plane resolution of 5 mm full-width at half maximum (FWHM). An intravenous line was inserted in the antecubital vein for [^11^C]-(+)-PHNO injection (radiosynthesis described in [33]). A transmission scan was acquired and the emission scan, acquired in 32-bit list mode over 90 minutes, began after bolus injection of [^11^C]-(+)-PHNO (mean ± SD, dose: 10.02±0.69 mCi; specific activity: 1206.45±448.37 mCi/µmol; mass: 2.31±0.77 µg). Emission data were reconstructed by 2D filtered back projection to yield dynamic images with 15 one-minute frames and 15 five-minute frames.

The cue-paradigm took place when the subject was lying down inside the PET camera and consisted of viewing a picture slideshow through a set of goggles (Icuiti Corporation, Rochester New York). All subjects viewed two different slideshows on separate occasions, in a randomized and counter-balanced order: either smoking-associated pictures (images of individuals smoking or smoking paraphernalia) [34], [35] or neutral pictures (everyday images, such as a window or a face of someone not smoking) [44]. These cues were shown to effectively induce craving by others [34], [35], [44]. This specific cue paradigm was validated in a set of six smokers meeting the same inclusion and exclusion criteria prior to completing this study and shown to elicit craving in 1-hour abstinent smokers (unpublished). A tactile component was also added in this cue paradigm; during the presentation of the smoking-associated cues, subjects were asked to hold a cigarette, and during the presentation of the neutral cues, a pen. This has previously been shown to elicit a greater craving response in conjunction with a visual cue paradigm [45]. The cues were presented in four ten-minute blocks with five-minute break intervals between blocks with an approximate total time of forty minutes of cue presentation (out of the 95 minute scan time). Each block consisted of approximately forty-five pictures, with three pictures being shown per seven seconds each followed by a fixation cross for twenty-one seconds. Following the final block, the subjects were asked to remain still until the end of the scan. [^11^C]-(+)-PHNO was injected after the first 10-minute block of cue presentation. The choice of injection time and length of imagery presentation was based on the work of Volkow and colleagues [6].

Subjective levels of craving and withdrawal symptoms were assessed when subject were placed into the scanner, before cue presentation (Pre-cue) and at scan completion (Final). Craving measures included a 21-item nicotine-specific Visual Analog Scale (VAS; 100 point continuous scale to score from agree to disagree or not at all to very much), the abbreviated Questionnaire of Smoking Urges (QSU) [46], the Tobacco Craving Questionnaire (TCQ) [47] and the Minnesota Nicotine Withdrawal Scale (MNWS) [48]. The VAS question “I have a craving for a cigarette” was also assessed at each five-minute break during the cue paradigm (Block 1–4) and every 15 minutes (twice, Block 5–6) for the rest of the scan.

On a separate day all subjects completed a proton density weighted MRI, acquired on a 1.5 T Signa-GE Scanner (TE = 13, TR>5300 ms, FOV = 22×22, 256×256, slice thickness = 2 mm, NEX = 2) for the purpose of region of interest (ROI) delineation.

### Data Analysis

Statistical Analysis. Differences in self-reported questionnaire measures and [^11^C]-(+)-PHNO regional binding between the two experimental conditions (neutral-cues vs. smoking-associated cues) were analyzed using repeated measures analysis of variance (RM-ANOVA). Paired sample t-tests were used for post-hoc comparisons. All analyses were two-tailed with an alpha level of 0.05. All tests were performed using the statistical software package SPSS, release 15.0 (SPSS Inc., Chicago IL, USA). Change in DA levels between the two experimental conditions was calculated as a percentage difference in binding potential (BP_ND_) obtained under neutral cues vs. smoking-associated cues. Correlations between change of DA and other outcome measures (such as expired CO and demographics) were calculated using Pearsons correlations. For measurement of craving during each experimental PET session, we measured the area under the curve (AUC) for the ratings obtained using the VAS question “I have a craving for a cigarette”. Absolute difference in AUC between the neutral and smoking cue conditions served as a single measure of cue-induced craving to be related to changes in BP_ND_ between the two experimental conditions.

#### PET Image Analysis

All PET images were analyzed using the automated image analysis software Regions of Mental Interest (ROMI) (details in [49]).

Bilateral sub-compartments of the striatum, including sensorimotor striatum (SMST), associative striatum (AST), and limbic striatum (LST) were automatically segmented [49] as described in [50]. The (whole) Globus Pallidus (GP) was delineated with the procedure previously described and validated [51]. Identification of midbrain grey matter voxels within the substantia nigra (SN) region was performed by using the the previously described automated procedure [49]. Cerebellar cortex (excluding vermis, lobules IX and X) served as reference region and has been previously described [49]. [^11^C]-(+)-PHNO time activity curves were obtained from dynamic data, and specific BP_ND_ was estimated in each ROI using the simplified reference tissue method (SRTM) [52]. Parameter estimation was performed with PMOD (Version 2.8.5; PMOD Technologies Ltd, Zurich, Switzerland).

## Results

### Subject Demographics

Out of 24 subjects that were determined to be eligible for the study, 18 completed the study. Two participants had a reaction to [^11^C]-(+)-PHNO (nausea), 3 did not feel comfortable with the scanning procedures and 1 tested positive for a pregnancy screen on the day of the scan. None of the participants that were included in the study tested positive for drugs of abuse on a gas chromatography and mass spectroscopy broad-spectrum urine toxicology drug screen. Subject demographics appear in Table 1.

**Table 1:** Descriptive characteristics for participants.

Descriptive	Mean ± Standard Deviation	p-value
Ratio Male:Female	10∶8	n/a
Age	37.4±7.1	n/a
Age of smoking initiation	15.8±2.5	n/a
Fagerstrom Test for Nicotine Dependence	6.2±1.7	n/a
Cigarettes per day (CPD)	18.2±6.0	n/a

	Smoking	Neutral	
Expired Carbon Monoxide	31.7±16.0	34.2±20.2	p>0.05
Mass Radioligand Injected (µg)	2.26±0.79	2.36±0.78	p>0.05
Corrected activity (mCi)	11.00±0.81	11.06±1.01	p>0.05
Specific Activity at time of injection (mCi/µmol)	1227.11±432.01	1185.79±475.78	p>0.05
Time since last cigarette (minutes)	74.28±15.77	75.00±15.33	p>0.05

### Craving and Withdrawal Measures

**Figure 1:**
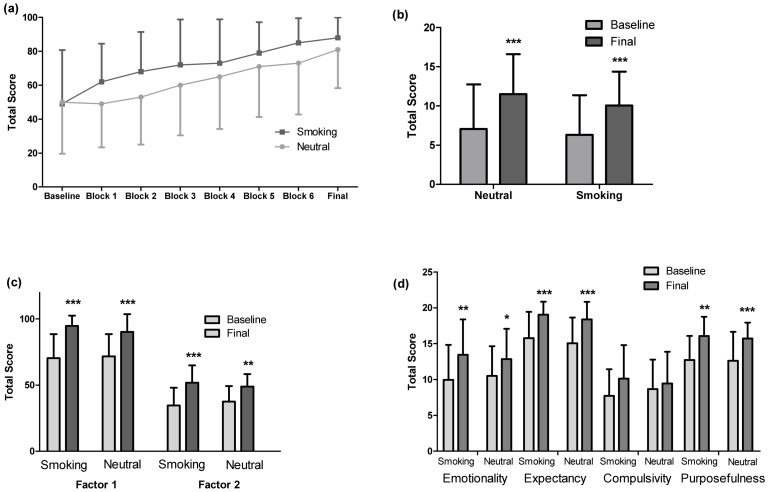
Craving measures assessed comparing smoking and neutral cue conditions. (a) Visual analog scale measurements of craving for a cigarette assessed every fifteen minutes during cue-presentation (Blocks 1–4) and every 15 minutes following the completion of the paradigm (Blocks 5–6). There was a main effect of cue type (Repeated Measures Analysis of Variance (RM-ANOVA, F_(1,17)_ = 6.935, p<0.05) and time (RM-ANOVA, F_(7,119)_ = 18.098, p<0.005) on craving for a cigarette. (b) Withdrawal symptoms as rated by the Minnesota Nicotine Withdrawal Scale at baseline (prior to cue presentation) and at the end of the scan. There was a main effect of time on withdrawal symptoms (RM-ANOVA, F_(1,17)_ = 23.996, p<0.001) (c) Cigarette craving as measured by the Questionnaire of Smoking Urges Factor 1 (urge to smoke for pleasure effects of smoking) and Factor 2 (urge to smoke for relief of negative effect of withdrawal) at baseline and at the end of the scan. There was a main effect of time on the urge to smoke for pleasurable effects (RM-ANOVA, QSU Factor 1 F_(1,17)_ = 53.866, p<0.001) and on the urge to smoke for relief of negative symptoms (RM-ANOVA, QSU Factor 2, F_(1,17)_ = 27.591, p <0.001) (d) Cigarette craving measured by the Tobacco Craving Questionnaire factors 1 (Emotionality), 2 (Expectancy), 3 (Compulsivity) and 4 (Purposefulness) at baseline and at the end of the scan. There was a main effect of time on the urge to smoke for relief of negative withdrawal effects (RM-ANOVA, TCQ 1/emotionality F_(1,17)_ = 11.907, p<0.005), urge to smoke for pleasurable effects (RM-ANOVA, TCQ 2/expectancy F_(1,17)_ = 25.510, p<0.001) and intention to smoke (RM-ANOVA, TCQ 4/purposefulness F_(1,17)_ = 14.336, p<0.005). * p<0.05, ** p<0.01, *** p<0.005

Subjective craving measures are illustrated in Figure 1. Repeated measures analysis of variance (RM-ANOVA) of craving scores obtained with the VAS question “I have a craving for a cigarette” indicated separate main effects of cue-type (F_(1,17)_  = 6.935, p<0.05) and time (F_(7,119)_ = 18.098, p<0.005) on self reported craving such that self-reported craving was greater during presentation of smoking vs. neutral cues and self reported craving increased with time (pre cue vs. block 2–6 all p<0.05). Time since last cigarette also significantly increased self-reported urge to smoke for pleasurable effects (QSU Factor 1 F_(1,17)_ = 53.866, p<0.001; TCQ 2/expectancy F_(1,17)_ = 25.510, p<0.001), urge to smoke for relief of negative withdrawal effects (QSU factor 2 F_(1,17)_ = 27.591, p<0.001; TCQ 1/emotionality F_(1,17)_ = 11.907, p<0.005,) intention to smoke (TCQ 4/purposefulness F_(1,17)_ = 14.336, p<0.005) and withdrawal symptoms (MNWS F_(1,17)_  = 23.996, p<0.001); however these measures were not different between conditions (all p>0.05). Exposure to smoking-related compared to neutral cues did not significantly increase self-reported withdrawal symptoms (MNWS), urge to smoke for pleasurable effects (QSU Factor 1 or TCQ 2), relief of negative effects (QSU Factor 2 or TCQ 1) or intention to smoke (TCQ 4).

### PET Findings

**Figure 2:**
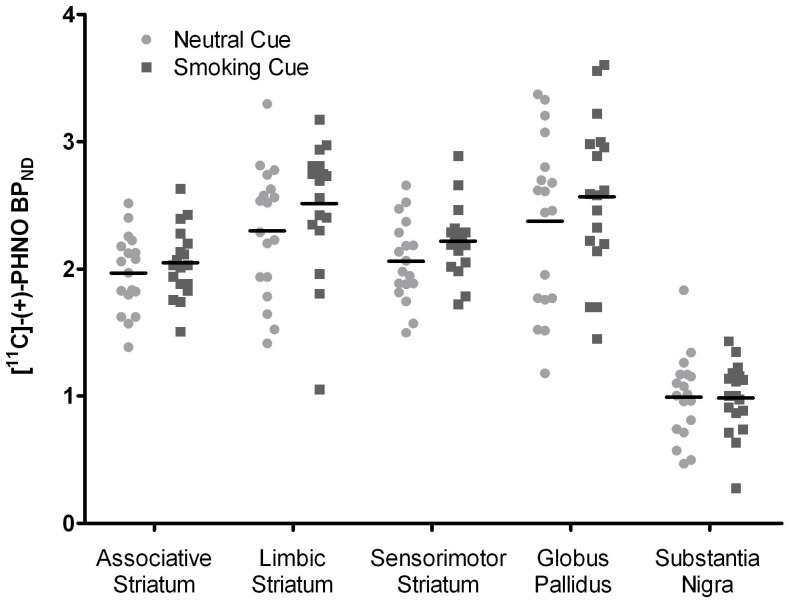
[^11^C]-(+)-PHNO BP_ND_ in brain regions of interest. [^11^C]-(+)-PHNO BP_ND_ in the associative striatum (AST), limbic striatum (LST), sensorimotor striatum (SMST), globus pallidus (GP) and substantia nigra (SN) in the neutral and smoking cue conditions. There were no overall significant differences between the cue conditions in [^11^C]-(+)-PHNO BP_ND_ in any of the apriori selected regions of interest.

RM-ANCOVA controlling for age and expired CO averaged over both conditions revealed no significant main effect of condition (i.e.: smoking vs neutral cues) (F_(1,15)_ = 0.053 p = 0.821) and interaction with regions of interest (AST, LST, SMST, GP and SN) (F_(4, 60)_ = 2.09, p = 0.99) (Figure 2). Overall [^11^C]-(+)-PHNO BP_ND_ during presentation of smoking cues was ∼6% greater when compared to presentation of neutral cues (Figure 2). There were no significant correlations between cue-induced craving (AUC using the repeated VAS measures) and % change in ROI binding from the neutral condition. There were also no correlations between any of the subjective self-report variables, demographic information (i.e.: severity of addiction), expired CO and regional cue-induced changes in binding.

**Figure 3:**
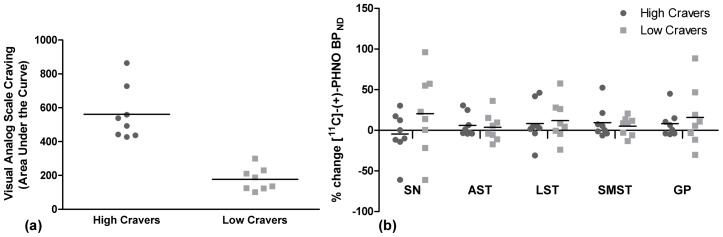
High cravers versus low cravers area under the visual analog scale curve and percent change in [^11^C]-(+)-PHNO BP_ND_ in regions of interest. (a) Area under the Visual Analog Scale curve in high cravers and low cravers. (b) Percent change in [^11^C]-(+)-PHNO BP_ND_ in the associative striatum (AST), limbic striatum (LST), sensorimotor striatum (SMST), globus pallidus (GP) and substantia nigra (SN) in high cravers versus low cravers. There were no significant differences in percent change in [^11^C]-(+)-PHNO BP_ND_ in any of the aprior regions of interest when comparing high cravers to low cravers (multivariate analysis of variance (MANOVA) F_(5,10)_ = 0.759, p = 0.731).

As a post-hoc analysis, a comparison of [^11^C]-(+)-PHNO BP_ND_ in high cravers (VAS AUC greater than sample mean, n = 8) vs. low cravers (VAS AUC lower than sample mean, n = 8) revealed no significant differences in percent change [^11^C]-(+)-PHNO BP_ND_ in any of the regions of interest (multivariate analysis of variance (MANOVA) F_(5,10)_ = 0.759, p = 0.731) (Figure 3). Two participants were not included in this analysis because they were within one standard deviation of the sample mean.

## Discussion

To our knowledge, this is the first study to assess fluctuation of DA levels in response to presentation of smoking-related cues compared to presentation of neutral cues in human smokers using PET. We found that presentation of smoking cues in our setting elicited craving on the VAS, but this finding was not supported by other measures of craving (QSU, TCQ). We also found that smoking cue presentation after a short (one hour) abstinence period did not result in changes in DA release as measured by changes in [^11^C]-(+)-PHNO BP_ND_.

In this cue paradigm, ratings of smoking-cue induced craving and of withdrawal increased over time in both experimental conditions. The VAS scale question “I have a craving for a cigarette”, which was repeatedly assessed throughout the cue presentation, revealed a significant increase in craving in the smoking-associated cue condition relative to the neutral condition (See Fig. 1A). However, we found that on most measures of craving, the increase was similar under both experimental conditions (See Fig. 1 B,C,D). Several factors may have impacted our ability to detect significant increases in craving on the QSU and TCQ. First, it is possible that the experimental conditions (subjects being placed in a PET scanner and pictures provided through goggles) may have created an unusual setting which was not conducive to natural situations in which cues may significantly induce craving for cigarettes. We did show in a pilot study that our smoking cues were capable of inducing craving relative to the neutral cues on the VAS in a sample of six participants (unpublished data) and other investigators have been able to induce craving with similar paradigms for a variety of drugs of abuse [6], [22], [25], [30], [31] including tobacco cigarettes [25], [28], [31]. A second possibility for the lack of difference between the two conditions on craving on the QSU and TCQ is that the plasma half-life of nicotine is on average 2 hours [53], and it is likely that there was still detectable levels of nicotine (in the plasma and also likely in the brain) of the participants during the sessions, minimizing craving and potentially the ability to detect cue-induce DA release. Thirdly, the duration of abstinence that we chose may have not been the optimal time point to capture changes in craving as assessed by the QSU and TCQ. Recent research has investigated the effect of abstinence time on smoking cue-induced craving and it was determined that smoking cue-induced craving increases with duration of abstinence [3]. Specifically, when comparing groups asked to remain abstinent for different periods of time (7 days, 14 days, 35 days) smoking cue-induced craving was greatest at 35 days of abstinence, suggesting an incubation of cue-induced craving [3]. It is of interest to note that in that study the subjects also did not display cue-induced craving after a short abstinence period.

Contrary to the research done in human studies of cocaine and opioid dependence [6], [21], [22], we were not able to detect significant differences in binding potential between the two conditions. A possible explanation could be related to the lack of difference we saw between conditions on craving scores as measured by the QSU and TCQ. Previous studies performed in cocaine users indicated that while analyzing the results based on the cravings scores, only the group displaying robust cravings scores had an associated elevation of DA levels in the striatal area [22]. However, we saw that even those participants that were the strongest cravers, as measured by the area under the VAS curve, did not show a significant percent change in [^11^C]-(+)-PHNO BP_ND_. Another possibility that could explain the lack of change in DA level in our paradigm could be related to the expectancy conditions that were chosen. DA neurons have been shown to respond to reward prediction error and under some conditions (stimuli presentation not resulting in the occurrence of the predicted reward) there may be decreases in DA cell firing [20]. Such phenomenon could account for the lack of elevation of DA, as measured by *in vivo* microdialysis, that has been reported under some conditions, such as contingent presentation of cocaine-associated stimuli in non-human primates[17]. The knowledge of occurrence of reward (e.g. possibility of smoking a cigarette) immediately following cue presentation could lead to strong activation of the reward pathway [25], [54] and it appears that glucose metabolism and activation (as measured by fMRI) in regions associated with reward is greatest when individuals are expecting to receive drug versus no expectation of drug [30], [54]. Collectively, this research suggests that the expectation of drug can influence the neurobiological reaction to drug or drug-associated cues. As participants in the current study were told that they would be able to smoke only one hour after exposure to the cue paradigm, this delay of accessing the reward after the cue presentation session may have contributed to an inability of our conditions to elicit elevation of DA levels. Another possibility for the lack of significant difference in [^11^C]-(+)-PHNO BP_ND_ between conditions is the fact that as subjects were likely under the influence of some nicotine, their dopaminergic system may have been already stimulated by nicotine and may have prevented the cue-induced craving to produce any further activation. This could mean that nicotine-induced DA release could have masked any cue-induced DA release. It is interesting to note that presence or absence of nicotine did not affect cue-elicited craving [55] or brain activation [43] in some studies, suggesting that some cravings or brain responses can be detected while nicotine is on board (however, see [25]).

We do not believe that the inability to detect an elevation of DA levels in our experiment is related to the choice of radiotracer. Although [^11^C]raclopride is perhaps the gold standard for measuring binding at D_2/3_ receptors, it is thought that [^11^C]-(+)-PHNO may be superior to measure acute fluctuations in synaptic DA release [36], [37]. When comparing DA-induced displacement of [^11^C]-(+)-PHNO and [^11^C]raclopride binding through a d-amphetamine challenge in anesthetized cats, there was approximately 83% inhibition of [^11^C]-(+)-PHNO binding potential compared to only approximately 56% of [^11^C]raclopride binding potential [37]. In a more recent direct comparison of ligands, DA release in the dorsal striatum, induced by an amphetamine challenge, was found to cause a 1.5 time greater reduction in [^11^C]-(+)-PHNO binding compared to [^11^C]raclopride binding [36]. However, [^11^C]-(+)-PHNO is not without limitations. Due to the sensitivity of [^11^C]-(+)-PHNO to detect small changes in DA release, it is also possible that it may have been more sensitive to the DA releasing effect of nicotine that may have been present after the short abstinence period. In addition, [^11^C]-(+)-PHNO also binds to D_3_ receptors, which can provide up to 100% of the binding signal in certain brain regions (substantia nigra), and [^11^C]-(+)-PHNO may therefore have a different sensitivity to detect DA release in these regions [56]. The current research did not find any differences in DA release between the two conditions (smoking vs. neutral), nor was there a greater magnitude of change in the D_3_ –enriched regions. [^11^C]-(+)-PHNO does act as an agonist at DA D_2_ and D_3_ receptors and previous studies have reported that pharmacological effects are present in approximately 14.3% of subjects scanned using this radiotracer [57]. Furthermore, it has been suggested that many studies utilizing [^11^C]-(+)-PHNO PET may use non-tracer doses [58]. Non-tracer doses may present a bias or underestimation of [^11^C]-(+)-PHNO BP_ND_, and therefore the mass dose effect should be taken in to consideration [36]. In the present study, although there was some variability in the specific activity at the time of injection within in each condition, we found no correlation between specific activity and [^11^C]-(+)-PHNO BP_ND_ in either of the conditions across any of the ROIs (p>0.05). Since we do not see a significant difference in the mass injected between the smoking and neutral cue conditions, we do not believe that the mass of radiotracer contributed to the negative findings of the current research. Finally, the test-retest reliability of [^11^C]-(+)-PHNO in the striatum ranges from approximately 2–7% [38]. We do not believe that the reason for an overall lack of main effect of condition on DA release was due to a lack of power to detect a difference (effect size  =  0.45).

This study showed no overall significant difference in DA levels between tobacco cue and neutral cue presentation conditions when measured at one-hour abstinence in human tobacco smokers. One limitation of this study is that under this short abstinence period, increases in craving were not detectable by the QSU and TCQ but there were significant increases on the repeated VAS throughout the cue paradigm. Further studies would need to explore the ability of tobacco cue presentation to elicit cravings and associated DA release after longer period of abstinence.
